# The Catechins Profile of Green Tea Extracts Affects the Antioxidant Activity and Degradation of Catechins in DHA-Rich Oil

**DOI:** 10.3390/antiox11091844

**Published:** 2022-09-19

**Authors:** Caroline Waingeh Nain, Eric Mignolet, Marie-France Herent, Joëlle Quetin-Leclercq, Cathy Debier, Melissa M. Page, Yvan Larondelle

**Affiliations:** 1 Louvain Institute of Biomolecular Science and Technology, UCLouvain, Croix du Sud, 4-5, L7.07.03, B-1348 Louvain-la-Neuve, Belgium; 2 Louvain Drug Research Institute, UCLouvain, Avenue Emmanuel Mounier, 72 bte B1.72.03, B-1200 Brussels, Belgium

**Keywords:** green tea extract, catechins, antioxidant activity, oxidative stability, catechin degradation, DHA-rich oil

## Abstract

This study investigated the effect of the catechins profile on the antioxidant activity of green tea extracts (GTEs) by comparing the antioxidant activity of an EGC-rich GTE (GTE1, catechin content: 58% EGC, 30.1% EGCG, 7.9% EC, and 3.9% ECG) and an EGCG-rich GTE (GTE2, catechin content: 60.6% EGCG, 17.7% EGC, 11.8% ECG, and 9.8% EC) in a DHA-rich oil. The effects of the individual catechins (EGC, EC, EGCG, and ECG) and reconstituted catechins mixtures (CatMix), prepared to contain the same amount of major catechins as in the GTEs, were also measured. All treatments (GTE1, CatMix1, GTE2, CatMix2, EGC250, EC250, EGCG250, and ECG250), each containing epistructured catechins at a concentration of 250 ppm, as well as the control (oil with no added antioxidant), were stored at 30 °C for 21 days with sampling intervals of 7 days. The antioxidant activity was assessed by measuring the peroxide value (PV) and p-anisidine value (p-AV) of oils. Changes in fatty acid content and catechins content were also monitored. Both GTEs enhanced the oxidative stability of the DHA-rich oil, but GTE1 demonstrated a stronger antioxidant activity than GTE2. No significant difference was observed between the PV of treatments with GTE1 and CatMix1 during storage, whereas the PV of oil with GTE2 was significantly higher than that with CatMix2 after 21 days. Among the individual catechins, EGC was the strongest antioxidant. Overall, the antioxidant activities of the extracts and catechins were observed in the decreasing order GTE1 ≈ EGC250 ≈ CatMix1 > GTE2 > EGCG250 ≈ CatMix2 > ECG250 > EC250. A significant change in fatty acid content was observed for the control and EC250 samples, and the catechins were most stable in GTE1-supplemented oil. Our results indicate that the EGC-rich GTE is a more potent antioxidant in DHA-rich oil than the EGCG-rich GTE.

## 1. Introduction

**Figure 1:**
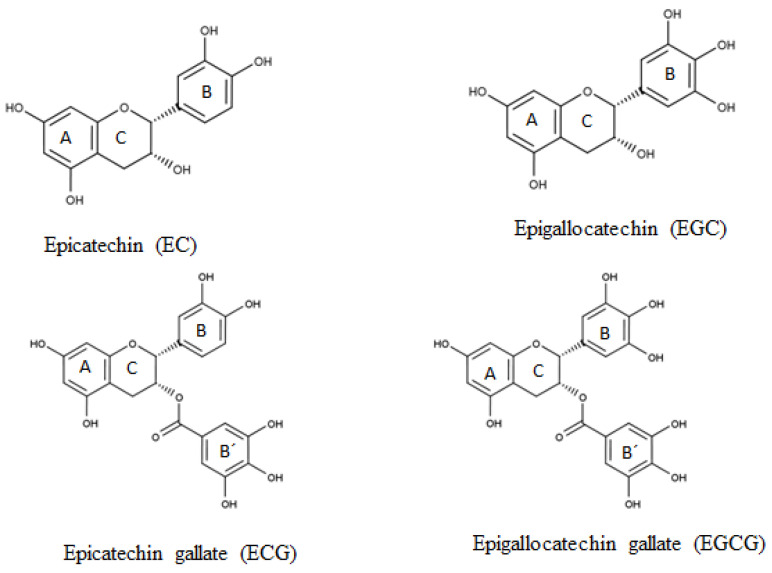
Chemical structure of major green tea catechins [17,19].

Green tea, prepared from the leaves of *Camellia sinensis*, is a widely consumed beverage rich in polyphenols. The interest in green tea has been further incited over the years by studies demonstrating its anti-carcinogenic [1,2], anti-microbial and antiviral [3,4], antihypercholesterolemic [5], anti-obesity [6,7], anti-inflammatory [8], and antioxidant properties [9,10]. The beneficial effects of green tea are attributed to its high catechins content, which accounts for about 15–27% of the dry weight of green tea leaves [11,12,13]. The major catechins in green tea are: (–)-epicatechin (EC), (–)-epigallocatechin (EGC), (–)-epicatechin gallate (ECG), and (–)-epigallocatechin gallate (EGCG), the latter being the most abundant [14]. The difference between these catechins is in the number of hydroxyl groups on the B-ring and the presence or not of a galloyl group on the C-ring (Figure 1). The major catechins can be converted during tea leave processing into their corresponding non-epistructured catechins, i.e., (±)-catechin, (–)-gallocatechin, (–)-catechin gallate and (–)-gallocatechin gallate [15,16]. Apart from catechins, tea leaves also contain small amounts of other phytochemicals such as caffeine, phenolic acids, and flavonols (mainly quercetin, kaempferol, myricetin, and rutin) [17,18].

The chemical structure of catechins seems to have an influence on their antioxidant activity, with the number and position of the hydroxyl groups in the molecule playing a substantial role [20]. Gallocatechins (EGC and EGCG) have a trihydroxyl group in the B-ring (pyrogallol group), while catechin gallates (ECG and EGCG) have an esterified gallate moiety attached to position 3 of the C-ring. The trihydroxyl group on the B-ring and the gallate moiety have both been associated with increased free radical scavenging ability of catechins, thus increased antioxidant activity [21]. Various studies have reported a stronger antioxidant activity for esterified catechins. He and Shahidi [22] observed the potency of catechins added at a concentration of 0.68 mmol/kg (200 mg EC equivalent/kg) in a fish meat system to be in the decreasing order EGCG ≈ ECG > EGC >> EC on the basis of peroxide value (PV). In another study, the antioxidant activity of catechins (200 ppm) in seal blubber and menhaden oils stored at 60 °C was in the decreasing order ECG > EGCG > EGC > EC [23]. In a β-carotene linoleate model system, the effectiveness was in the order ECG > EGCG = EC > EGC with respect to β-carotene bleaching [24]. However, in some studies, a stronger antioxidant activity was recorded for catechins containing the pyrogallol moiety. The antioxidant activity of tea catechins (200 ppm) in canola oil heated at 95 °C for different times varied in the decreasing order EGC > EGCG > EC > ECG [25]. Huang and Frankel (1997) observed varying trends in the antioxidant activity of tea catechins in different lipid systems. In corn oil triglycerides stored at 50 °C, the antioxidant potency of catechins (140 µM) was observed in the order EGC ≈ EGCG ≈ ECG > EC. In contrast, all the catechins were prooxidants in a corresponding corn oil-in-water emulsion, whereas in soy lecithin liposomes also stored at 50 °C, EGCG was the best antioxidant followed by EC, EGC, and ECG at 20 µM [26]. These authors suggested that variation in the antioxidant activities among catechins could be partly explained by differences in their reducing potential, stability, and relative partition between phases in different lipid systems [26].

Green tea extracts (GTEs) rich in catechins are produced from green tea leaves by different processes such as conventional solvent extraction, ultrasound-assisted extraction, microwave-assisted extraction, high-pressure processing, or supercritical fluid extraction, among others [27]. The amount of catechins recovered in an extract can vary depending on the extraction solvent, temperature, and duration [28,29]. Green tea leaves contain as most abundant catechin EGCG (40–69%), followed by EGC (12–23%), ECG (13–21%), and EC (5–9%) [12,30,31], but these proportions could also vary with environmental conditions, variety, as well as agronomic conditions such as plucking time and leaf age [13,32]. For example, Chen et al. [33] measured higher levels of EGCG and ECG in young leaves compared to mature leaves, while old leaves were richer in EGC and EC. This implies that catechins profiles of GTEs would vary with respect to the source and age of tea leaves and the extraction procedure, which could affect antioxidant effectiveness.

This study compares the effect of two GTEs with different catechins profiles, one rich in EGC (GTE1) and the other rich in EGCG (GTE2), on the oxidative stability of a DHA-rich oil. To ascertain that the antioxidant effect is a result of catechins (epistructured) and not due to other active compounds in the GTE, we also evaluated the antioxidant activity of reconstituted catechins mixtures in the proportions present in the GTEs, as well as the effect of the individual catechins. In addition, the fate of catechins in the oil was also assessed while assessing possible degradation products. This study contributes to a better understanding of the role of the catechins profile in the antioxidant activity of GTEs, with the ultimate aim to guide the choice of GTE that will better enhance the oxidative stability of oils and derived products enriched in highly valuable fatty acids.

## 2. Materials and Methods

### 2.1. Materials 

The two GTEs were both products of Naturex (Avignon, France). Green tea leaf PE 50% Poly.UV/20% Catec.HPLC/7% EGCG (EA830365): powdered extract of green tea leaves, standardized to a minimum content of 50% polyphenols, 20% catechins, and 7% EGCG, referred to as GTE1 in this study and green tea extract PE 95% Poly.UV/60% Catec.HPLC/30% EGCG (ED840370): powdered extract of green tea leaves, standardized to a minimum content of 95% polyphenols, 60% catechins and 30% EGCG., referred to as GTE2. 

DHA-rich oil with no added antioxidants was kindly provided by KD Pharma (Bexbach, Saarland, Germany). The oil had a total polyunsaturated fatty acid (PUFA) content of 906.8 mg/g with a mean content of 661.5 mg/g being docosahexaenoic acid (DHA) and 137.4 mg/g of eicosapentaenoic acid (EPA). 

The analytical standards, EC, EGC, ECG, and EGCG with the following respective HPLC purity: ≥99%, ≥98%, ≥97.5%, and ≥98%, were purchased from Extrasynthese (Genay, France). All other chemicals used in this study were either of GC-grade, HPLC-grade, or analytical grade.

### 2.2. Quantification of Major Catechins in GTE

The catechins profiles of GTE1 and GTE2 were determined by measuring their content in EGC, EC, EGCG, and ECG as previously described [34]. GTE (0.3 g) was weighed in a centrifuge tube to which 15 mL of absolute ethanol was added. After vortexing for 1 min to solubilize the extract, the mixture was sonicated (70W, 40 kHz) for 15 min at room temperature and centrifuged for 5 min at 1800× *g* (Eppendorf Centrifuge 5810 R, Hamburg, Germany). Various dilutions of the ethanolic extract were prepared in a 1% (*v*/*v*) formic acid in water solution and filtered through a 0.45 µm syringe filter into HPLC injection vials. The HPLC conditions used for this analysis were as we previously described [34]. The catechins were identified and quantified by comparing retention times and peak areas to those of a pure standard mixture of EGC, EC, ECGC, and ECG, prepared at different concentrations to obtain calibration curves. The analysis was repeated four times for each extract, and the results are expressed in mg/g of extract.

### 2.3. Oxidative Stability Test

The antioxidant effects of GTE1 and GTE2 were evaluated in a DHA-rich oil under accelerated aging at 30 °C during a storage period of 21 days. GTE1 was added to the oil at a concentration of 1000 ppm (250 ppm of major catechins), which was the most effective concentration in our previous study [34], and GTE2 was incorporated into the oil at a concentration of 450 ppm to ensure a total catechin content of 250 ppm as in GTE1-supplemented samples. To evaluate the contribution of major catechins to the antioxidant effect of extracts, oil samples were also supplemented with reconstituted mixtures of catechins in the same proportions present in GTE1 (CatMix1) and GTE2 (CatMix2). The effect of individual catechins (EGC, EC, EGCG and ECG) were also evaluated at a concentration of 250 ppm. The GTEs and catechins were added to the oil as an ethanolic solution. Each GTE powder was precisely weighed in a centrifuge tube and solubilized in absolute ethanol by vortexing for 1 min and sonicating for 15 min. The quantity of ethanol used did not exceed 4% weight of the final oil mixture. As for the reconstituted catechins mixtures, EGC, EC, EGCG, and ECG powders were each dissolved in ethanol to obtain solutions with a concentration of 5 mg/mL. Combinations of these catechins solutions were then prepared to obtain catechins profiles similar to GTE1 and GTE2 (Table 1). The oil and antioxidant blends were mixed by stirring with a glass rod for 3 min and flushed with nitrogen for 10 min to evaporate the ethanol. Control samples were prepared in the same way as the treatments except without any added catechins. The oil samples were then distributed (10 g) in amber bottles (30 mL, Ø 2.7 cm), and the opened bottles were stored in an oven at 30 °C for 21 days with a sampling interval of 7 days (day 0, 7, 14, and 21). All samples were prepared in triplicate. The oxidative status of oils was determined on the sampling day by measuring the PV and para-anisidine value (*p-AV*), and the remaining oil was flushed with argon and stored at −20 °C for subsequent analyses of catechins and fatty acid contents.

### 2.4. Evaluation of Oxidative Status

The PV, which measures primary oxidation products, was determined by iodometric titration according to the AOAC official method 965.33 [35]. The *p-AV* was measured according to the AOCS official method Cd 18–19 [36]. *p-AV* is a measurement of secondary oxidation products generated during the decomposition of hydroperoxides, primarily 2-alkenals and 2,4-alkadienals.

### 2.5. Quantification of the Catechins Content in Oil Samples

Catechins were extracted from oil samples supplemented with GTE and quantified as previously described [34]. To extract catechins, 0.5 g of oil sample was mixed with 1 mL of 90% ethanol in a 1.5 mL Eppendorf tube, vortexed for 1 min and centrifuged for 5 min at 20,000× *g*. The ethanolic phase (supernatant) was transferred into a 2 mL Eppendorf tube, and a second extraction of the remaining oil phase was carried out. The supernatants from the two extractions were combined and the ethanol was evaporated under vacuum in a SpeedVac concentrator Savant SPD121P (ThermoFisher Scientific, Asheville, NC, USA). The residue was solubilized in 1 mL or 2 mL of 1% (*v*/*v*) formic acid in water depending on the expected concentration of catechins and filtered through a 0.45 µL syringe filter into an HPLC vial. The samples were then analyzed using the reverse-phase HPLC conditions described earlier. The results are expressed in µg/g of oil.

### 2.6. Identification of Catechins Degradation Products 

To identify catechins degradation products detected on the reverse-phase HPLC chromatograms of oil treatments, the catechins extracts from these treatments were analyzed by high-performance liquid chromatography-electrospray ionization-high resolution-mass spectrometry (HPLC-ESI-HR-MS). The HPLC-ESI-HR-MS system consisted of an HPLC equipped with a degasser, a pump, an autosampler, and a PDA detector, and this was connected to a Thermo Instruments ESI-MS system (LTQ-Orbitrap-XL mass spectrometer, Thermo Fisher Scientific, Bremen, Germany). The HPLC column used was a Phenomenex Kinetex 2.6 µm PFP 100A column (150 mm × 4.6 mm, and the HPLC conditions were the same as previously described [34]. The MS was operated both in the positive ion mode in a mass range of 100–2000 m/z and in a negative ion mode. In positive ion mode, the MS conditions were the following: sheath gas flow rate: 10 au (arbitrary unit), auxiliary gas flow rate: 5 au, sweep gas flow rate: 5 au, capillary temperature: 265 °C, capillary voltage: 15 V, tube lens: 90 V and I spray voltage: 3 kV. In negative ion mode, the MS conditions were the following: sheath gas flow rate: 10 au (arbitrary unit), auxiliary gas flow rate: 10 au, sweep gas flow rate: 10 au, capillary temperature: 275 °C, capillary voltage: −35 V, tube lens: −110 V and I spray voltage: 5 kV.

### 2.7. Quantification of Fatty Acids

The fatty acid composition of the samples was analyzed at the start of the storage test (day 0) and the end of the test (day 21) using the method previously described by Mellery et al. [37]. The fatty acids in oil were first converted to fatty acid methyl esters (FAME) via a 2-step methylation with KOH and HCl with nonadecanoic acid (C19: 0, Sigma-Aldrich) used as an internal standard. The FAMEs were then extracted in hexane and separated by gas chromatography. Fatty acids in the samples were identified and quantified by comparing the retention times and peak areas with a standard mixture of 43 pure FAME standards (Larodan, Solna, Sweden). Chromatograms were processed using Chromeleon 7 (ThermoFisher Scientific, Waltham, MA, USA), and the results are expressed as mg/g of oil.

### 2.8. Statistical Analysis

All experiments were performed in triplicate, and results are expressed as mean ± standard deviation. The data for the oxidative status and catechins degradation were subjected to a one-way or two-way analysis of variance (ANOVA), and means were compared between treatments using the Tukey–Kramer HSD (honestly significant difference) test. Differences in means between the fatty acid contents on day 0 and day 21 for each treatment was achieved by the Student’s *t*-test. All statistical analyses were performed using JMP Pro version 16 (SAS institute inc., Cary, NC, USA), and the differences between the means were considered significant at *p* ≤ 0.05. 

## 3. Results

### 3.1. Catechins Content of Green Tea Extracts

**Figure 2:**
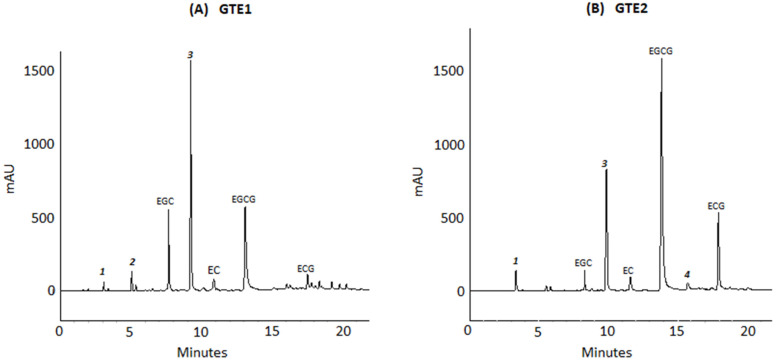
Chromatographic separation of catechins from two green tea extracts, GTE1 (**A**) and GTE2 (**B**). A binary solvent gradient system with solvent A (1% formic acid in water) and solvent B (1% formic acid in acetonitrile) was used. Identified and quantified compounds: EGC = epigallocatechin, EC = epicatechin, EGCG = epigallocatechin gallate, ECG = epicatechin gallate. Other compounds 1 = gallic acid, 2 = gallocatechin, 3 = caffeine, 4 = gallocatechin gallate.

The catechins contents of GTE1 and GTE2 analyzed by HPLC are shown in Table 1. The catechins of interest were the epistructured catechins, generally considered the major green tea catechins. The total content of these catechins in GTE1 represented 25.3% (*w*/*w*) of the extract, with the most abundant catechins being EGC accounting for 58% of the quantified catechins, followed by EGCG (30%), EC (7.9%), and ECG (3.9%). In contrast, GTE2 contained 55.8% (*w*/*w*) of total catechins in the following order of abundance EGCG (60.6%) > EGC (17.7%) > ECG (11.8%) > EC (9.8%). Therefore, the total catechins content of GTE2 was 2.2 times higher than that of GTE1. Apart from the quantified catechins, other peaks were visible in the GTE chromatograms, identified as gallic acid (1), gallocatechin (2), caffeine (3), and gallocatechin gallate (4) (Figure 2).

### 3.2. Oxidative Status of Oil during Storage

#### 3.2.1. Peroxide Value

**Figure 3:**
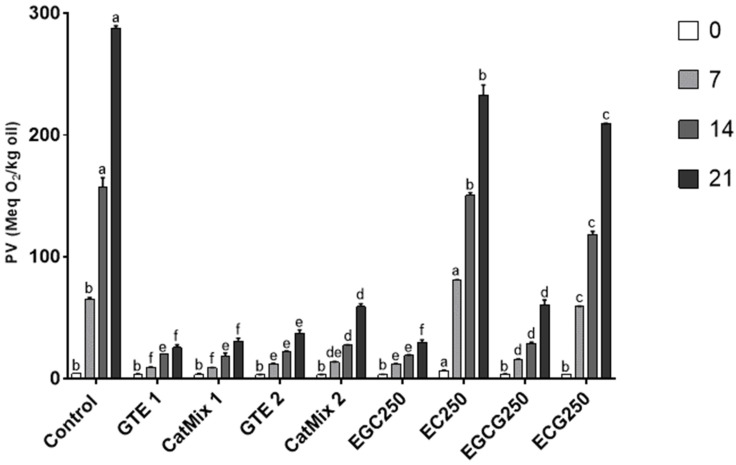
Effect of green tea extracts (GTEs) and catechins on the peroxide value (PV) of DHA-rich oil during a 21-day storage at 30 °C. Results are expressed as mean ± standard deviation, n = 3. Control = DHA-rich oil, GTE1 = DHA-rich oil + 1000 ppm GTE1, CatMix1 = DHA-rich oil + reconstituted catechin mixture of GTE1, GTE2 = DHA-rich oil + 450 ppm GTE2, CatMix2 = DHA-rich oil + reconstituted catechin mixture of GTE2, EGC250, EC250, EGCG250 and ECG250 = DHA-rich oil + 250 ppm of EGC or EC or EGCG or ECG, respectively. Significant differences (*p* ≤ 0.05) are represented by different lower-case letters (a–f) among treatments at a specific storage time.

The PV of the DHA-rich oil was 3 meq O_2_/kg at the start of the storage test, and the increase rate during the 21 days of storage at 30 °C varied with respect to the treatment. As shown in Figure 3, incorporating GTE1 and GTE2 into the oil slowed the formation of hydroperoxides. The PV of the control sample increased by 95-fold in 21 days, which was significantly different (*p* < 0.0001) from the 9-fold and 12-fold increases observed for treatments with GTE1 and GTE2, respectively. No significant difference was detected between the PV of oils supplemented with GTE1 and GTE2 from day 0 to day 14 of storage, but by day 21, the GTE1-supplemented oil had a significantly lower (*p* = 0.02) PV than the oil supplemented with GTE2. This indicates a more persistent antioxidant activity for GTE1 than for GTE2.

To understand the contribution of the major catechins to the antioxidant activity of GTE, the effect of GTE1 and GTE2 on PV was compared to the reconstituted catechins mixtures (Figure 3). There was no significant difference between the PV of treatments with GTE1 and its reconstituted catechins mixture, CatMix1, throughout the storage period, although a slightly lower PV was recorded for GTE1-supplemented samples (PV of 25.5 ± 2.3 meq O_2_/kg oil) compared to CatMix1 (30.9 ± 2.3 meq O_2_/kg) after 21 days of storage. On the other hand, the PV of GTE2-supplemented oil (37.5 ± 2.4 meq O_2_/kg oil) was significantly lower (*p* < 0.0001) than that of oil supplemented with CatMix2 (59.1 ± 2.4 meq O_2_/kg oil) after 21 days of storage, with no significant differences observed from day 0 to day 14. 

The effect of individual catechins on the PV of the DHA-rich oil was also tested, and the results are also presented in Figure 3. The PVs of oils supplemented with individual catechins were significantly lower than the control. The PV increase was significantly higher for EC250 than for the other catechins-supplemented samples throughout the storage period, whereas EGC250 resulted in a significantly lower PV than the other treatments. EGC250 had, thus, a more potent effect on preventing PV increase than EGCG250, which is consistent with the observation that GTE1 rich in EGC (58% of major catechins) is also more effective than GTE2 rich in EGCG (60% of major catechins). 

Comparing the change in PV among the samples rich in EGC and those rich in EGCG, we observed no significant differences among the PV of treatments with GTE1, CatMix1, and EGC250. Accordingly, there was no significant difference between the PV of oils supplemented with CatMix2 and EGCG250. Therefore, the most abundant individual catechins in each of these treatments appears to be responsible for the majority of the antioxidant activity.

#### 3.2.2. *p*-Anisidine Value

The *p-AV* is a measure of the accumulation of secondary oxidation products, such as aldehydes, that arise from the breakdown of hydroperoxides. As shown in Table 2, the *p-AV* of the control sample increased significantly from 2.8 ± 0.6 to 100.6 ± 8.2 over the 21-day storage period. The incorporation of the GTEs and catechins slowed the formation of secondary products. A decrease or no significant change was observed in the *p-AV* of samples containing GTE1, CatMix1, GTE2, CatMix2, EGC250, and EGCG250 during the storage, whereas samples supplemented with EC250 and ECG250 showed a significant increase in *p-AV*. The *p-AV* of the ECG250 condition remained lower than the control throughout the storage period, whereas the difference was significant only on days 7 and 14 for the EC250 condition. These results are in accordance with the PV results, as samples that recorded higher PVs also revealed an increase in the *p-AV*. 

The overall effect of treatments in improving the oxidative status of oil during storage was in the decreasing order GTE1 ≈ EGC250 ≈ CatMix1 > GTE2 > EGCG250 ≈ CatMix2 > ECG250 > EC250.

### 3.3. Changes in Fatty Acid Content of Oil during Storage

The DHA-rich oil used in this study had a PUFA content of 906.8 mg/g with the major PUFAs being DHA (C22:6c4,c7,c10,c13,c16,c19) at 661.5 mg/g and EPA (C20:5c5,c8,c11,c14,c17) at 137.4 mg/g. The sum of monounsaturated fatty acids in the oil was 7.9 mg/g, while saturated fatty acids were 1.2 mg/g of oil.

**Figure 4:**
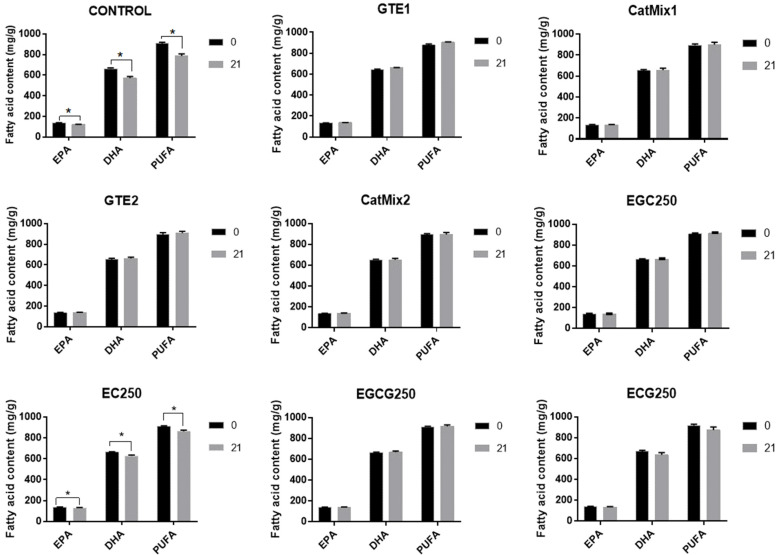
Effect of green tea extracts (GTE) and catechins on the eicosapentaenoic acid (EPA), docosahexaenoic acid (DHA) and total polyunsaturated fatty acid (PUFA) content of a DHA-rich oil after 21 days of storage at 30 °C. Results are expressed as mean ± standard deviation, n = 3. A significant difference between pairs is indicated by a *. Control = DHA-rich oil, GTE1 = DHA-rich oil + 1000 ppm GTE1, GTE2 = DHA-rich oil + 450 ppm GTE2, CatMix1 = DHA-rich oil + reconstituted catechins mixture of GTE1, CatMix2 = DHA-rich oil + reconstituted catechins mixture of GTE2, EGC250 = DHA-rich oil + 250 ppm EGC, EC250 = DHA-rich oil + 250 ppm EC, EGCG250 = DHA-rich oil + 250 ppm EGCG, ECG250 = DHA-rich oil + 250 ppm ECG.

Figure 4 shows the effect of the different catechins treatments on EPA, DHA, and total PUFA content of the DHA-rich oil after 21 days of storage. The control oil showed a significant decrease in EPA (*p* = 0.0023), DHA (*p* = 0.0019), and total PUFA (*p* = 0.0018), with a 12.4%, 13.6%, and 13.2% reduction, respectively. The supplementation of DHA-rich oil with GTE1, GTE2, and their respective catechins mixtures prevented any loss in fatty acids during the storage period, which was also true for EGC250 and EGCG250 treatments. A non-significant reduction in EPA, DHA, and total PUFA, of 4%, 4.9%, and 4.6%, respectively, was observed for the oil with ECG250, whereas the oil with EC250 recorded a significant decrease in these fatty acids, with a reduction of 5% for EPA (*p* = 0.0065), 5.4% for DHA (*p* = 0.0065), and 5.2% for total PUFA (*p* = 0.0063). These results are consistent with the changes observed in the *p-AV* of the oil samples.

### 3.4. Depletion of Catechins

**Figure 5:**
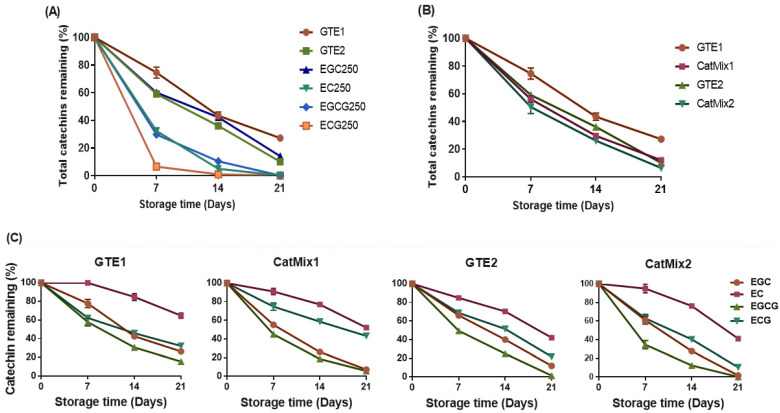
Kinetics of catechins depletion in DHA-rich oil supplemented with green tea extracts, catechins mixtures or individual catechins. (**A**) Depletion of total catechins from GTE-supplemented and catechins-supplemented oil samples; (**B**) comparing depletion of total catechins in GTE and CatMix-supplemented oil samples; (**C**) depletion of individual catechins present in oil supplemented with GTEs and their reconstituted mixtures. GTE1 = DHA-rich oil + 1000 ppm GTE1, GTE2 = DHA-rich oil + 450 ppm GTE2, CatMix1 = DHA-rich oil + reconstituted catechins mixture of GTE1, CatMix2 = DHA-rich oil + reconstituted catechins mixture of GTE2, EGC250 = DHA-rich oil + 250 ppm EGC, EC250 = DHA-rich oil + 250 ppm EC, EGCG250 = DHA-rich oil + 250 ppm EGCG, ECG250 = DHA-rich oil + 250 ppm ECG.

All treatments were prepared to contain about 250 µg/g (250 ppm) of total catechins, equivalent to 1000 ppm of GTE1 and 450 ppm of GTE2. The activity of the catechins in the oil started immediately upon addition, as their depletion was already evident in extracts from day 0 oil samples (Table S1). The level of depletion of these catechins during the storage period varied with respect to treatment (Figure 5A–C). After 7 days of storage, the oil supplemented with GTE1 had the highest residual total catechins content of 186 µg/g (74.5% of initial content). The residual total catechins in the other treatments was as follows: EGC250 with 150 µg/g (60.1% initial content), GTE2 with 147.8 µg/g (59.1% initial content), EC250 with 80.9 µg/g (32.3% initial content), EGCG250 with 74.5 µg/g (29.8% initial content), and ECG250 with 16.25 µg/g (6.5% initial content) (Figure 5A). By the end of the 21-day storage period, the catechins in EC250, EGCG250, and ECG250 treatments were almost completely depleted, while GTE1, EGC250, and GTE2 samples still had 27.2%, 14.1%, and 10.1% residual catechins, respectively. A slower loss in catechins was, therefore, observed for the GTE1 supplemented samples, while the fastest depletion was recorded for the ECG250 condition. 

Figure 5B shows the residual catechins in oils supplemented with GTE and CatMix. The depletion rate of catechins was higher in CatMix samples compared to their corresponding GTE counterparts, suggesting an effect of the other polyphenolic compounds and caffeine present in the GTEs. After the first 7 days of storage, the catechins remaining in the CatMix1-supplemented oil were at 56.1% of the initial content, which was significantly different (*p* = 0.0053) from the 74.5% residual content in the GTE1 condition. After 21 days, a significantly lower value (*p* = 0.0016) was still evident for CatMix1. As for CatMix2 and GTE2 samples, total residual catechins contents of 50.4% and 59.1%, respectively, were recorded after 7 days of storage, and a significant difference (*p* = 0.0265) was observed after 21 days of storage with a residual content of 6.4% and 10.1%, respectively.

The level of depletion of the individual catechins in oil samples supplemented with GTE1, CatMix1, GTE2, and CatMix2 are shown in Figure 5C. The least depleted catechins in these treatments was EC, with the remaining content after 21 days being 13.0 ± 0.6 µg/g (64.9% of initial content) for GTE1, 10.4 ± 0.5 µg/g (52.2% of initial content) for CatMix1, 10.1 ± 0.4 µg/g (42.1% of initial content) for GTE2, and 9.9 ± 0.3 µg/g (41% of initial content) for CatMix2. On the flip side, EGCG was the fastest depleted compound, with almost none left in GTE2 (1.5%) and CatMix2 (0.8%) after 21 days, while 16.6% and 5.7% of the initial content was left in treatments with GTE1 and CatMix1, respectively. The absolute concentrations of the catechins in the oil during storage are presented as supplementary data (Table S1). The depletion of the individual catechins in the GTE and CatMix samples was in the decreasing order of EGCG > EGC > ECG > EC, irrespective of the initial individual catechins concentrations in the samples.

### 3.5. Degradation Product of Epigallocatechin Gallate or an Intermediate Product in Its Antioxidant Activity?

**Figure 6:**
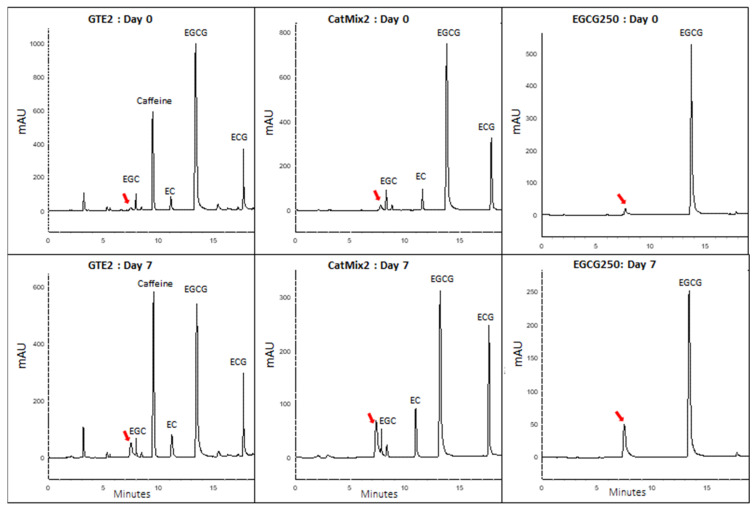
HPLC-UV chromatograms of a degradation product formed in catechins-supplemented DHA-rich oils during an accelerated storage test at 30 °C. GTE2 = DHA-rich oil + 450 ppm GTE2, CatMix2 = DHA-rich oil + reconstituted catechins mixture of GTE2, EGCG250 = DHA-rich oil + 250 ppm EGCG. The extracts from EGCG250-supplemented oil injected on HPLC were two times more diluted than the GTE2 and CatMix2 samples to avoid peak saturation. The degradation product is indicated by the arrow.

The HPLC chromatograms of catechins extracted from the oil treatments supplemented with GTE2, CatMix2, and EGCG250 revealed the presence of an unexpected peak that elutes at a retention time of 7.65 min (Figure 6), but this peak was not detected in the other treatments (Figure S1). Since this peak was not present in the chromatogram of GTE2 (Figure 2B) nor the chromatograms of EGC250, EC250, and ECG250 oil samples, we postulated that this compound was a degradation product of EGCG, which resulted from its reaction in the oil. Thus, we quantified the new product as an EGCG equivalent (Table 3). This product was detected in small amounts in day 0 oil samples and increased after seven days at 30 °C before showing a decreasing trend. The product was more abundant in the oil with EGCG250, which could be explained by the higher depletion level of EGCG in this treatment (Figure 5). The depletion of this new product was slower in oil samples supplemented with GTE2 compared to the EGCG250 and CatMix2 treatments.

Analysis by HPLC-ESI-HR-MS to identify this compound was futile as no signal was observed on MS both in the positive and negative electron ionization modes. The absence of a signal on MS could be due to the low concentration of this product in our samples or poor ionization characteristics. 

## 4. Discussion

The antioxidant activity of catechins (flavan-3-ols) and GTEs has been demonstrated in various food matrices, including pastry, meat products, table spreads, food emulsions, and bulk oils [38,39,40,41,42]. The effectiveness of catechins as antioxidants is attributed to their ability to donate hydrogen atoms to free radicals, thus acting as free radical scavengers [20]. Catechins also slow oxidation by acting as metal ion chelators, binding metal ions such as iron and copper, which otherwise initiate the oxidation process [43]. In our previous study, our results indicate that GTE was more effective in improving the oxidative stability of a DHA-rich oil than α-tocopherol [34]. Investigating the activity of GTE1 and GTE2 in the present study confirmed the ability of these two GTEs to delay the oxidation of a DHA-rich oil. However, GTE1 resulted in a lower PV in the oil compared to GTE2 after 21 days at 30 °C. Since the oil samples were prepared to contain a similar total catechins content, the difference in the protective effect of GTE1 and GTE2 could be attributed to the difference in their catechins profiles. In a related study, Toschi et al. [44] evaluated the antioxidant ability of three GTEs in a refined peanut oil subjected to forced oxidation at 98 °C and concluded that the two GTEs with higher levels of EGCG and ECG were more effective antioxidants than the less concentrated extract. However, the concentration of total catechins added in the peanut oil samples was not similar at the start of the experiment, thus suggesting that the differences observed may be explained by the difference in concentration of catechins rather than the catechins composition of the GTEs, as is the case in the present study.

The higher antioxidant activity observed for GTE1 could be associated with its high EGC content (58% of the total catechins), given that the EGC250 samples recorded the lowest PV compared to oils supplemented with the other individual catechins. Indeed, the ability of the individual catechins to slow oxidation was in the order EGC250 > EGCG250 > ECG250 > EC250. This result is similar to that reported by Chen and Chan [25], who reported an antioxidant activity of catechins in the decreasing order EGC > EGCG > EC > ECG when incorporated at a concentration of 200 ppm in canola oil heated at 95 °C. Moreover, in line with our results, Huang and Frankel [26] investigated the ability of catechins to inhibit hydroperoxide formation in corn oil oxidized at 50 °C and observed the effectiveness in the order EGC ≈ EGCG ≈ ECG > EC. Contrary to our results, some studies have reported a higher antioxidant activity for EGCG compared to EGC. For instance, the antioxidant activity of catechins was in the order EGCG ≈ ECG > EGC >> EC in ground mackerel cooked at 75 °C and stored for 7 days at 4 °C [22]. Lee et al. [13] investigated the antioxidant activity of pure catechins by their ability to scavenge 2,2′-azino-bis-3-ethylbenzthiazoline-6-sulphonic acid (ABTS) and 1,1-diphenyl-2-picrylhydrazyl (DPPH) radicals and reported antioxidant activity in the order EGCG ≥ GCG ≥ ECG > EGC ≥ GC ≥ EC ≥ C. The free radical scavenging ability of catechins is partly related to their reduction potential, which indicates the ease with which the catechins will donate a hydrogen atom to a free radical and the stability of the resulting antioxidant radical [45]. EGC and EGCG have the lowest reduction potentials (0.43V) compared to ECG (0.55V) and EC (0.57V), suggesting they are superior electron donors [45]. In addition, the antioxidant activity of catechins is also associated with the presence of the trihydroxyphenyl B-ring and/or a galloyl group on the C-ring, with the trihydroxyphenyl B-ring acting as the principal site of antioxidant reactions in EGCG and EGC [46]. 

Comparing the effect of GTEs with their reconstituted catechins mixtures, we observed no significant difference in the ability to delay hydroperoxide formation between GTE1 and CatMix1. Therefore, the antioxidant activity of GTE1 could mostly be attributed to the epistructured catechins. On the other hand, GTE2 acted as a better antioxidant than CatMix2 after 21 days at 30 °C, indicating that other compounds present in the extract (other polyphenols and caffeine) exhibited antioxidant activity or acted in synergy with the major catechins. Similarly, a previous study reported a higher antioxidant activity of a crude GTE than its reconstituted mixture of individual catechins in a β-carotene linoleate model system [24]. Nonetheless, the crude GTE with a total catechins content of 300 mg/g made up of 17.2% (EC), 39.2% (EGC), 7.6% (ECG), and 36% (EGCG) was different from the GTEs used in this study, which contained higher levels of either EGC (GTE1) or EGCG (GTE2). Gallocatechin gallate, present in GTE2, might contribute to its antioxidant effectiveness since it has been reported to possess a similar antioxidant activity to EGCG when subjected to an anti-2,2-diphenyl-1-picrylhydrazyl (DPPH) free radical assay, whereas gallocatechin, was shown to be less potent as an antioxidant [47]. In another study, the results of online DPPH radical-scavenging activity tests of a mixed catechins standard showed that the antioxidant activity of catechins decreased in the order EGC > gallic acid > EGCG > EC > ECG > catechin, while caffeine had almost no DPPH-scavenging activity [48]. The presence of gallic acid in GTE2 could thus also contribute to its antioxidant activity and an enhanced protective effect compared to CatMix2. Caffeine though inert to DPPH radicals, is effective in scavenging hydroxyl radicals and has also been shown to inhibit lipid peroxidation [49,50]. Thus, the presence of caffeine in the extracts might also enhance the antioxidant activity.

The incorporation of GTEs in the DHA-rich oil used in the current study prevented the degradation of fatty acids, hence no change in DHA and EPA content was observed in GTE-supplemented oils during the 21-day storage, whereas a 12 to 13% decrease was observed in the control sample. The fatty acid content of the other treatments remained relatively stable, apart from EC250 and ECG250 treatments, which recorded a decrease in EPA and DHA content. Yin et al. [51] observed no change in the DHA content of a mixed antioxidant-supplemented microalgal DHA-rich oil (44.37% DHA) subjected to accelerated oxidation at 60 °C for 10 days, but a decrease of 4.35% was observed after 20 days of oxidation. In another recent study, the DHA content of purified algal oil stored for two days at 60 °C significantly decreased from 35.3% to 31.6%, which equals a 10.5% decrease [52]. In the present study, a decrease in fatty acid was only observed for treatments with PVs > 200 meq O_2_/kg oil and *p-AV*s > 50, which may imply that a change in fatty acid content is only evident when oxidation is at a very advanced stage. 

As the catechins protected the oil from oxidation, they were depleted in the process. The rate of depletion of the total catechins varied among treatments in the decreasing order ECG250 > EC250 ≈ EGCG250 > GTE2 > EGC250 > GTE1. As free radical scavengers, catechins donate hydrogen from the hydroxyl group, thus breaking the auto-oxidation chain. The resulting unpaired electron in the catechins is delocalized within the ring structure, resulting in a relatively unreactive free radical, which forms an o-quinone, followed by reactions such as oligomerization and intramolecular rearrangement [53]. Catechins losses could be due to the combined effect of oxidation, degradation, epimerization, and polymerization during storage, with contributing factors being temperature and oxygen availability [54]. The depletion of the individual catechins in the GTE and CatMix-supplemented oils was observed in the order EGCG > EGC > ECG > EC. Catechins with higher antioxidant activity (EGCG and EGC) were depleted faster, and this could be linked to their lower reduction potential, which makes hydrogen abstraction easier. Wang and Zhuo [55] incorporated GTE in bread and observed losses in catechins during the breadmaking process in the order EGC > EGCG > ECG > EC. The catechins depletion rate was higher for the GTE-supplemented samples than for the CatMix samples, which might indicate that other polyphenolic compounds and caffeine present in the extracts play a protective role on the catechins. It is worth noting that the pattern of depletion of individual catechins, when added singly to the oil, differed from when added as a mixture. 

Although all the catechins recorded losses during storage, a product of degradation was only detected for EGCG, which could indicate that the HPLC method used was not suitable for detecting the degradation products of the other catechins or the concentrations were too low to be detected. The depletion of EGCG resulted in a new peak that eluted at 7.65 min, just prior to the elution of EGC on the chromatogram. This unidentified product was more polar than EGCG, given that on reverse-phase HPLC, more polar compounds are eluted first. Furthermore, since this molecule eluted very close to EGC, it is reasonable to assume that it is similar in structure or polarity to EGC, possibly derived from an EGCG by loss of its gallic acid group. In a previous study, Dai et al. [56] observed a product ion of m/z 305 after thermal treatment at 100 °C of a solution containing EGCG. They concluded that this product resulted from a loss of a gallic acyl group from EGCG. Kondo et al. [57] suggested that the scavenging effect of EGCG on peroxyl radicals in liposomal and aqueous systems resulted in the conversion of EGCG to an anthocyanin-like compound with cleavage of the galloyl moiety. Contrarily, all three oxidation products of EGCG derived from its reaction with peroxyl radicals, which were identified and isolated by Valcic et al. [46,58], resulted from changes in the B-ring of EGCG rather than the galloyl moiety. The EGCG product observed in the present study decreased over time, which may suggest that it is an intermediate breakdown product or a product that also possesses antioxidant activity.

## 5. Conclusions

This study confirmed the effect of GTEs and catechins as natural antioxidants that can retard the oxidation of highly unsaturated oils. The results of this study also indicate that the catechins profile of GTEs has an impact on their antioxidant effectiveness in DHA-rich oil. Both GTE1 and GTE2 enhanced the oxidative stability of the respective supplemented DHA-rich oil. However, GTE1, containing a higher proportion of EGC, was more effective in delaying oxidation than GTE2 rich in EGCG. The epistructured catechins (EGC, EC, EGCG, and ECG) were shown to be responsible for the antioxidant activity of GTE1, as no significant difference was observed between the antioxidant activity of GTE1 and CatMix1 during the storage period. In contrast, the effect of GTE2 was more prominent than that of CatMix2, suggesting that apart from the epistructured catechins, other compounds in GTE2 contributed to its antioxidant activity. Among individual catechins, EGC demonstrated a stronger antioxidant activity in the DHA-rich oil. Overall, the antioxidant activity of the GTEs and catechins were observed in the decreasing order GTE1≈ EGC250 > GTE2 > EGCG250 > ECG250 > EC250. A significant reduction in fatty acid content was observed for the control and EC250-supplemented oils, and the catechins were most stable in GTE1-supplemented oil. These findings indicate that the EGC-rich GTE is a more potent antioxidant in DHA-rich oil than the EGCG-rich GTE.

## Figures and Tables

**Table 1:** Mean content of epistructured catechins in an EGC-rich green tea extract (GTE1) and an EGCG-rich green tea extract (GTE2).

	GTE1		GTE2	
Catechins	Absolute (mg/g Extract)	Relative (% Total Catechins)	Absolute (mg/g Extract)	Relative (% Total Catechins)
Epigallocatechin (EGC)	147.2 ± 1.4	58.0	99.2 ± 4.1	17.7
Epicatechin (EC)	20.0 ± 0.9	7.9	54.7 ± 2.8	9.8
Epigallocatechin gallate (EGCG)	76.4 ± 7.9	30.1	338.8 ± 25.5	60.6
Epicatechin gallate (ECG)	10.0 ± 3.4	3.9	66.2 ± 5.2	11.8
Total	253.6 ± 11.2	100.0	558.9 ± 37.7	100.0

Results are expressed as mean ± standard deviation of four repeated analyses of each green tea extract.

**Table 2:** Changes in the *p*-anisidine value (*p*-AV) in DHA-rich oil supplemented or not with green tea extracts or catechins during storage at 30 °C for 21 days.

	*p*-AV			
Treatment	Day 0	Day 7	Day 14	Day 21
Control	2.8 ± 0.6 ^aD^	15.9 ± 2.8 ^aC^	47.9 ± 1.5 ^aB^	100.6 ± 8.2 ^aA^
GTE1	0.0 ± 0.0 ^bA^	0.0 ± 0.0 ^cA^	0.3 ± 0.5 ^deA^	0.32 ± 0.3 ^cA^
CatMix1	0.0 ± 0.0 ^bA^	0.0 ± 0.0 ^cA^	0.0 ± 0.0 ^eA^	0.0 ± 0.0 ^cA^
GTE2	3.3 ± 0.5 ^aA^	0.0 ± 0.0 ^cC^	1.2 ± 0.8 ^deB^	0.0 ± 0.0 ^cC^
CatMix2	2.9 ± 0.78 ^aA^	1.1 ± 0.9 ^cAB^	2.1 ± 1.0 ^deA^	0.0 ± 0.0 ^cB^
EGC250	3.2 ± 0.5 ^aA^	1.3 ± 1.2 ^cB^	3.6 ± 0.5 ^dA^	0.0 ± 0.0 ^cC^
EC250	3.5 ± 0.1 ^aD^	13.2 ± 3.9 ^abC^	28.0 ± 1.8 ^bB^	93.1 ± 6.4 ^aA^
EGCG250	2.6 ± 0.4 ^aA^	1.2 ± 1.1 ^cA^	3.2 ± 2.2 ^deA^	0.0 ± 0.0 ^cB^
ECG250	3.4 ± 0.3 ^aC^	8.9 ± 1.7 ^bC^	15.3 ± 1.1 ^cB^	69.6 ± 4.2 ^bA^

Results are expressed as mean ± standard deviation, n = 3. Significant differences (*p* ≤ 0.05) within a column are represented by different lower-case letters (a–e) and by different uppercase letters (A-D) within a row. Control = DHA-rich oil, GTE1 = DHA-rich oil + 1000 ppm GTE1, CatMix1 = DHA-rich oil + reconstituted catechin mixture of GTE1, GTE2 = DHA-rich oil + 450 ppm GTE2, CatMix2 = DHA-rich oil + reconstituted catechin mixture of GTE2, EGC250, EC250, EGCG250 and ECG250 = DHA-rich oil + 250 ppm of EGC or EC or EGCG or ECG, respectively.

**Table 3:** Changes in the content of an EGCG degradation product during the storage of a DHA-rich oil supplemented with a green tea extract rich in EGCG (GTE2), a corresponding catechins mixture (CatMix2) or pure EGCG (EGCG250) at 30 °C for 21 days.

Storage Time (Days)	Concentration (µg EGCG Equivalent/g Oil)		
	GTE2	CatMix2	EGCG250
0	3.2 ± 0.2 ^c^	4.5 ± 0.7 ^b,c^	10.7 ± 0.6 ^b,^*
7	11.7 ± 0.8 ^a^	13.1 ± 1.8 ^a^	27.0 ± 3.1 ^a,^*
14	9.3 ± 1.0 ^b^	7.2 ± 1.2 ^b^	14.3 ± 3.7 ^b,^*
21	8.1 ± 1.0 ^b,^*	2.1 ± 0.3 ^c^	2.1 ± 0.2 ^c^

Values are mean ± SD (n = 3). Significant differences (*p* ≤ 0.05) within columns are represented by different lower-case letters (a-c). * indicates a significant difference (*p* ≤ 0.05) between treatments at a specific storage time (within row). GTE2 = DHA-rich oil + 450 ppm GTE2, CatMix2 = DHA-rich oil + reconstituted catechin mixture of GTE2, EGCG250 = DHA-rich oil + 250 ppm EGCG. The day 0 and 7 extracts from the EGCG250-supplemented oil injected on HPLC were two times more diluted than GTE2 and CatMix2.

## Data Availability

Data are contained within the article.

## Supplementary Materials

The following supporting information can be downloaded at: https://www.mdpi.com/article/10.3390/antiox11091844/s1. Table S1: Changes in the concentration (µg/g) of catechins in DHA-rich oil supplemented with green tea extracts, catechins mixtures or individual catechins during storage at 30 °C for 21 days.; Figure S1: HPLC-UV chromatograms of catechins extracted from DHA-rich oils supplemented with GTE or catechins during accelerated ageing at 30 °C.
