# Supplementary material for: The Catechins Profile of Green Tea Extracts Affects the Antioxidant Activity and Degradation of Catechins in DHA-Rich Oil
Source: Antioxidants (Basel). 2022 Sep 19;11(9):1844. doi: 10.3390/antiox11091844 (PMC9495874; doi:10.3390/antiox11091844)
Supplement: Supplementary file 1 [file antioxidants-11-01844-s001.zip › antioxidants-1911416-supplementary.pdf]

# Supplementary materials

## The Catechins Profile of Green Tea Extracts Affects the Antioxidant Activity and Degradation of Catechins in DHA-Rich Oil

**Caroline Waingeh Nain[1], Eric Mignolet[1], Marie-France Herent[2], Joëlle Quetin-Leclercq[2], Cathy Debier[1], Melissa M Page[1] and Yvan Larondelle[1*]**

[1] Louvain Institute of Biomolecular Science and Technology, UCLouvain, Croix du Sud, 4-5, L7.07.03, B-1348 Louvain-la-Neuve, Belgium

[2] Louvain Drug Research Institute, UCLouvain, Avenue Emmanuel Mounier, 72 bte B1.72.03, B-1200 Brussels, Belgium

* Correspondence: yvan.larondelle@uclouvain.be.

**Table S1**: Changes in the concentration (μg/g) of catechins in DHA-rich oil supplemented with green tea extracts, catechins mixtures or individual catechins during storage at 30°C for 21 days.

| Treatment | | The concentration of catechins (μg/g oil) | | | | | |
|---|---|---|---|---|---|---|---|
| | | **Storage day** | **EGC** | **EC** | **EGCG** | **ECG** | **Total** |
| GTE1 | **Added** | | **147.2** | **20** | **76.4** | **10** | **253.6** |
| | Extracted | 0 | 146.2 ± 7.6 | 20.5 ± 2.0 | 57.9 ± 4.2 | 6.6 ± 0.5 | 231.1 ± 13.4 |
| | | 7 | 114.8 ± 6.1 | 20.6 ± 0.6 | 44.3 ± 3.1 | 6.3 ± 0.2 | 186.0 ± 9.4 |
| | | 14 | 62.9 ± 4.1 | 17.0 ± 0.8 | 23.3 ± 1.4 | 4.6 ± 0.1 | 107.9 ± 6.0 |
| | | 21 | 39.3 ± 1.1 | 13.0 ± 0.6 | 12.6 ± 0.6 | 3.2 ± 0.1 | 68.1 ± 2.4 |
| CatMix1 | **Added** | | **145.8** | **20** | **75** | **10** | **250.8** |
| | Extracted | 0 | 107.6 ± 6.2 | 17.7 ± 1.0 | 47.9 ± 2.2 | 7.6 ± 0.3 | 180.8 ± 9.6 |
| | | 7 | 80.1 ± 3.5 | 18.2 ± 0.7 | 34.3 ± 1.4 | 7.5 ± 0.4 | 140.1± 5.6 |
| | | 14 | 37.3 ± 1.4 | 15.4 ± 0.2 | 14.0 ± 0.5 | 5.9 ± 0.2 | 72.6 ± 1.8 |
| | | 21 | 10.4 ± 2.8 | 10.4 ± 0.5 | 4.3 ± 0.9 | 4.3 ± 0.2 | 29.4 ± 4.4 |
| GTE2 | **Added** | | **44.6** | **24.6** | **152.5** | **29.8** | **251.6** |
| | Extracted | 0 | 38.3 ± 1.1 | 22.4 ± 0.3 | 132.2 ± 4.5 | 24.4 ± 0.9 | 217.3 ± 6.5 |
| | | 7 | 31.1 ± 1.0 | 20.7 ± 0.8 | 75.9 ± 2.3 | 19.9 ± 0.3 | 147.6 ± 4.3 |
| | | 14 | 17.6 ± 0.7 | 17.5 ± 0.6 | 37.9 ± 1.1 | 14.9 ± 0.2 | 88.0 ± 1.3 |
| | | 21 | 6.3 ± 0.8 | 10.1 ± 0.4 | 2.2 ± 0.6 | 6.6 ± 0.3 | 25.2 ± 1.8 |
| CatMix2 | **Added** | | **44.6** | **24.6** | **152.4** | **29.8** | **251.5** |
| | Extracted | 0 | 35.6 ± 1.0 | 22.7 ± 0.6 | 101.8 ± 4.2 | 21.2 ±0.7 | 181.2 ± 6.4 |
| | | 7 | 28.8 ± 1.8 | 24.0 ± 1.4 | 55.1 ± 5.7 | 18.2 ± 1.3 | 126.1 ± 10.2 |
| | | 14 | 12.2 ± 0.2 | 18.3 ± 0.5 | 18.9 ± 1.8 | 11.7 ± 0.3 | 61.1 ±2.6 |
| | | 21 | 0.7 ± 0.2 | 9.9 ± 0.3 | 1.3 ± 0.1 | 4.0 ± 0.1 | 15.9 ± 0.4 |
| EGC250 | **Added** | | **250.0** | | | | **250.0** |
| | Extracted | 0 | 203.4 ± 2.1 | - | - | - | 203.4 ± 2.1 |
| | | 7 | 150.3 ± 1.7 | | - | - | 150.3 ± 1.7 |
| | | 14 | 105.6 ± 3.4 | - | - | - | 105.6 ± 3.4 |
| | | 21 | 35.1 ± 3.9 | - | - | - | 35.1 ± 3.9 |

| | | | | | | | |
|---|---|---|---|---|---|---|---|
| EC250 | **Added** | | **-** | **250.0** | **-** | **-** | **250.0** |
| | Extracted | 0 | - | 220.6 ± 1.1 | - | - | 220.6 ± 1.1 |
| | | 7 | - | 80.9 ± 1.5 | - | - | 80.9 ± 1.5 |
| | | 14 | - | 12.4 ± 2.0 | - | - | 12.4 ± 2.0 |
| | | 21 | - | 0.4 ± 0.01 | - | - | 0.4 ± 0.01 |
| EGCG250 | **Added** | | **-** | **-** | **250.0** | **-** | **250.0** |
| | | 0 | - | - | 179.9 ± 3.4 | - | 179.9 ± 3.4 |
| | Extracted | 7 | - | - | 74.6 ± 3.1 | - | 74.6 ± 3.1 |
| | | 14 | - | - | 26.0 ± 1.9 | - | 26.0 ± 1.9 |
| | | 21 | - | - | 0.3 ± 0.02 | - | 0.3 ± 0.02 |
| **ECG250** | **Added** | | **-** | **-** | **-** | **250.0** | **250.0** |
| | Extracted | 0 | - | - | - | 175.3 ± 8 | 175.3 ± 8 |
| | | 7 | - | - | - | 16.3 ± 1.0 | 16.3 ± 1.0 |
| | | 14 | - | - | - | 2.4 ± 0.03 | 2.4 ± 0.03 |
| | | 21 | - | - | - | 0.2 ± 0.1 | 0.2 ± 0.1 |

EGC = epigallocatechin, EC = epicatechin, EGCG = epigallocatechin gallate, ECG = epicatechin gallate. Control = DHA-rich oil, GTE1 = DHA-rich oil + 1000 ppm GTE1, GTE2 = DHA-rich oil + 450 ppm GTE2, CatMix1 = DHA-rich oil + reconstituted catechins mixture of GTE1, CatMix2 = DHA-rich oil + reconstituted catechins mixture of GTE2, EGC250 = DHA-rich oil + 250 ppm EGC, EC250 = DHA-rich oil + 250 ppm EC, EGCG250 = DHA-rich oil + 250 ppm EGCG, ECG250 = DHA-rich oil + 250 ppm ECG.

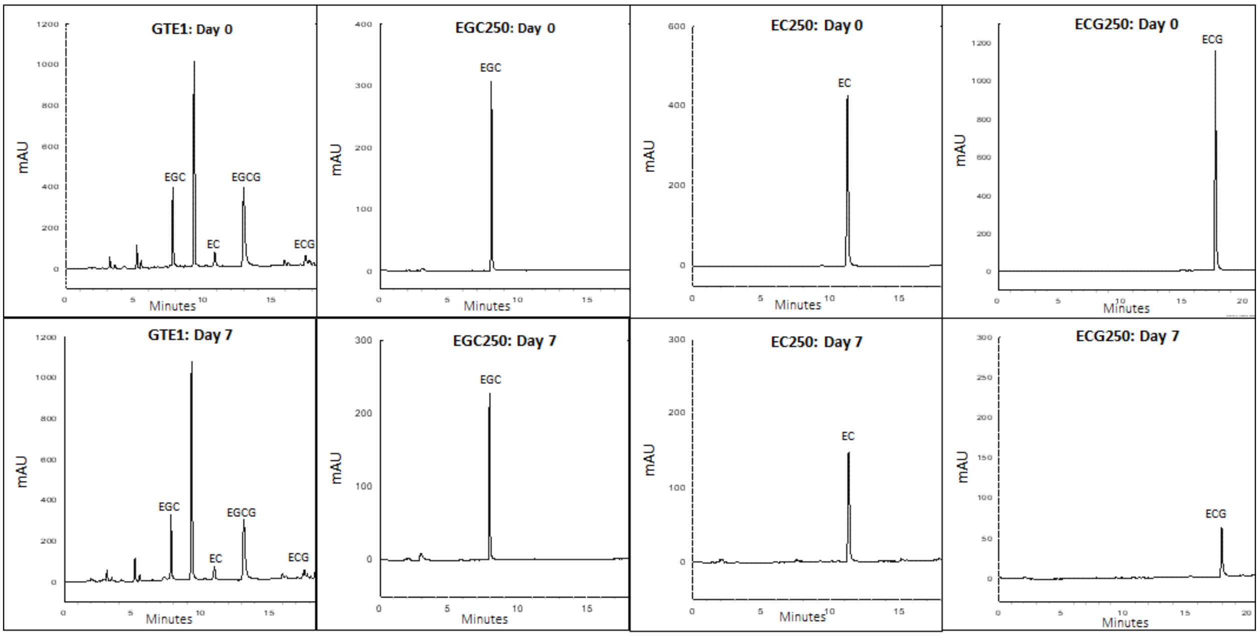


**Figure S1**. HPLC-UV chromatograms of catechins extracted from DHA-rich oils supplemented with GTE or catechins during accelerated ageing at 30°C. EGC = epigallocatechin, EC = epicatechin, EGCG = epigallocatechin gallate, ECG = epicatechin gallate. GTE1 = DHA-rich oil + 1000 ppm GTE1, EGC250 = DHA-rich oil + 250 ppm EGC, EC250 = DHA-rich oil + 250 ppm EC, ECG250 = DHA-rich oil + 250 ppm ECG. The degradation product at a retention time of 7.65 min was not detected in these samples.
